# Common olfactory ensheathing glial markers in the developing human olfactory system

**DOI:** 10.1007/s00429-016-1313-y

**Published:** 2016-10-07

**Authors:** Karen Oprych, Daniel Cotfas, David Choi

**Affiliations:** 1 Department of Brain, Repair and Rehabilitation, Institute of Neurology, University College London, Queen Square, London, WC1N 3BG UK; 2 National Hospital for Neurology and Neurosurgery, Queen Square, London, WC1N 3BG UK

**Keywords:** OEC, Olfactory ensheathing cells, Human, Olfactory bulb, Foetal, Immunocytochemistry

## Abstract

The in situ immunocytochemical properties of olfactory ensheathing cells (OECs) have been well studied in several small to medium sized animal models including rats, mice, guinea pigs, cats and canines. However, we know very little about the antigenic characteristics of OECs in situ within the adult and developing human olfactory bulb and nerve roots. To address this gap in knowledge we undertook an immunocytochemical analysis of the 11–19 pcw human foetal olfactory system. Human foetal OECs in situ possessed important differences compared to rodents in the expression of key surface markers. P75^NTR^ was not observed in OECs but was strongly expressed by human foetal Schwann cells and perineurial olfactory nerve fibroblasts surrounding OECs. We define OECs throughout the 11–19 pcw human olfactory system as S100/vimentin/SOX10+ with low expression of GFAP. Our results suggest that P75^NTR^ is a robust marker that could be utilised with cell sorting techniques to generate enriched OEC cultures by first removing P75^NTR^ expressing Schwann cells and fibroblasts, and subsequently to isolate OECs after P75^NTR^ upregulation in vitro. O4 and PSA-NCAM were not found to be suitable surface antigens for OEC purification owing to their ambiguous and heterogeneous expression. Our results highlight the importance of corroborating cell markers when translating cell therapies from animal models to the clinic.

**Electronic supplementary material:**

The online version of this article (doi:10.1007/s00429-016-1313-y) contains supplementary material, which is available to authorized users.

## Introduction

For over two decades researchers have been studying the therapeutic potential of olfactory ensheathing cells (OECs) to treat traumatic injuries to the central nervous system (Barnett and Riddell 2007; Mackay-Sim and St John 2011; Raisman et al. 2012; Gladwin and Choi 2015). OECs are unique glial cells that exist exclusively within the olfactory system and perform a vital role in supporting the continual turnover of short-lived olfactory receptor neurons (ORNs) throughout adult life (Doucette 1993). New ORNs must accurately extend their axons from their origin in the olfactory epithelium, along the peripheral olfactory nerves, and into the central nervous system where they must form appropriate connections within the olfactory nerve layer of the olfactory bulb. The olfactory system is the only location in humans where such axon growth and path-finding occurs continuously across the peripheral-central nervous system boundary throughout life (Doucette 1991). The ensheathment of ORN axons by processes of OECs along the entire olfactory pathway is vital for this to occur. OECs are dynamic and versatile glia uniquely possessing characteristics of both peripheral Schwann cells and central nervous system astrocytes. These properties enable OECs to maintain both the functioning of mature ORNs and promote and support new ORN growth in both the peripheral and central nervous system.

With this exceptional combination of properties, OECs are prime candidates for therapeutic transplants to promote CNS regeneration. To date, hundreds of studies in experimental nervous system injury models and recent studies in humans have provided convincing evidence to support the clinical development of OEC transplantation (Mackay-Sim et al. 2008; Granger et al. 2012; Tabakow et al. 2014; Watzlawick et al. 2016). These studies have shown OECs can provide a wide range of therapeutic effects. OEC transplants have been reported to enhance tissue preservation and promote functional recovery by; reducing inflammation and lesion cavitation; promoting neuronal survival and plasticity; promoting angiogenesis; enhancing remyelination of spared axons, and promoting regeneration of damaged axonal pathways by bridging the lesion with a growth-permissive substrate (Keyvan-Fouladi et al. 2003; Lakatos et al. 2003; Khankan et al. 2016). A recent meta-analysis of 62 experimental studies in rodent models of SCI concluded that OEC transplants enhanced functional recovery with an effect size of 19.2 % (after adjusting for publication bias and missing data) measured using the Basso, Beattie, and Bresnahan locomotor scoring method (Watzlawick et al. 2016).

In experimental transplantation studies OECs are most often obtained from the olfactory nerve layer (ONL) of the olfactory bulb (OB), and less commonly from the olfactory mucosa. OECs can be isolated from adult or embryonic/foetal tissue and expanded to a limited degree in culture. Commonly, OEC transplants contain a mixture of cells depending on the tissue source, however despite the potential complexity of the cell mixture, often only details of the OEC and olfactory fibroblast components are described in published papers. The majority of our knowledge of OEC biology and reparative properties has arisen from rodent studies. In particular, the immunocytochemical characteristics of OECs have been well studied in rats and mice with the general consensus that OECs express the low affinity neurotrophin receptor P75^NTR^, S100 and GFAP in situ and in vitro (Gong et al. 1994; Franceschini and Barnett 1996; Au et al. 2002). These three antigens are the most commonly used markers to identify OECs in therapeutic cultures (Kawaja et al. 2009).

To improve culture yields, rodent OECs can be purified from mixed cell cultures or enriched using various methods, most commonly by cell sorting using the expression of P75^NTR^. This can be performed on the initial cell suspension obtained after tissue digestion, or after plating and culturing for several days (Kawaja et al. 2009). One drawback to this method is that contaminating Schwann cells present in peripheral nerve bundles in the mucosa and surrounding arterioles in the ONL also express P75^NTR^ and so, may be co-isolated along with OECs. Due to similarities in their antigenic characteristics, currently there are no established protocols for purifying rodent OECs from Schwann cells. Studies in larger animal models have revealed inter-species differences in the antigenic characteristics of OECs in situ (Wewetzer et al. 2011). In a canine model, P75^NTR^ was expressed in non-myelinating Schwann cells and in connective tissue surrounding olfactory nerves in situ, but not in OECs (Bock et al. 2007). As canine OECs only upregulate P75^NTR^ after a period of time in culture, Schwann cells can be removed from the initial cell digest using P75^NTR^ immunopanning (Ziege et al. 2013). The resulting suspension can be cultured to allow time for OECs to upregulate P75^NTR^ and then subjected to a second round of immunopanning to isolate OECs from any remaining contaminating cells. These studies highlight the importance of corroborating OEC markers when extrapolating protocols developed in rodent models to other species. A lack of P75^NTR^ expression in OECs in situ was also reported in cats and guinea pig (Smithson and Kawaja 2009). Immunocytochemical characterisation in situ not only provides markers for purification and identification of OECs, but also provides insights into their complex biology. Studies in rodents have demonstrated OECs differentially express several antigens in the olfactory bulb and mucosa, and between the inner and outer layers of the olfactory bulb (Vincent et al. 2005). OECs in the olfactory nerves and outer ONL express P75^NTR^, whilst OECs in the inner ONL do not express P75^NTR^ but do express neuropeptide Y and GFAP (Franceschini and Barnett 1996; Au et al. 2002). These data suggest functional differences may exist between anatomical OEC populations. Indeed, studies have shown OECs from different regions of the ONL and olfactory nerve differ in their phagocytic capabilities, migratory and cell–cell interactive behaviours (Lohr et al. 2014; Ekberg and St John 2015). These differential properties could be exploited to enhance the efficacy of therapeutic transplants.

The immunocytochemical characterisation of human OECs in situ has generally been limited to the analysis of biopsies of the adult human olfactory mucosa; very little is known about the antigenic characteristics of adult and developing human OECs within the olfactory bulb and nerve roots. The small number of studies that have described human OECs in situ in the adult olfactory mucosa have presented conflicting results with some suggesting OECs do express P75^NTR^ (Bianco et al. 2004; Kachramanoglou et al. 2013; Winstead et al. 2014), whilst Holbrook et al. (2016) suggest that similar to canines, olfactory perineurial cells express P75^NTR^, but OECs do not (Holbrook et al. 2016). Validation of P75^NTR^ expression or lack thereof in human OECs in situ is necessary for developing protocols for correctly identifying and enriching OECs in cultures for future clinical trials.

For clinical application, autologous OEC transplants would be ideal however, obtaining OECs from the olfactory nerve layer of a patient requires an invasive and risky brain operation resulting in the unilateral loss of an entire olfactory bulb and risks of anosmia, meningitis, stroke or death. Biopsies of olfactory mucosa are a safer alternative but isolating OECs from these biopsies can be difficult and yields can be variable, especially where a patient may possess a pre-existing nasal disease (Kachramanoglou et al. 2013). Alternatively, in the future OECs may be generated from patient stem cells such as hair follicle neural crest stem cells or from induced pluripotent stem cells. However, despite some recent progress, our knowledge of the pathways that control OEC development from the neural crest, and how these pathways differ from Schwann cell development are limited (Barraud et al. 2010; Miller et al. 2016). Thus, additional allogeneic sources of OECs may be required until appropriate differentiation protocols to generate OECs from patient stem cells have been developed and validated.

Despite ethical considerations, discarded human foetuses could provide an additional source of human olfactory tissue for research purposes and a supplemental cell source during the initial clinical development of OEC therapies. Transplants of human foetal cells have been used historically to provide proof-of-concept for various regenerative cell therapies such as photoreceptor replacement therapy (Humayun et al. 2000). Foetal OECs could be used clinically where it is either not possible to obtain patient autologous OECs due to pre-existing pathologies or where patient autologous OEC cultures are not of sufficient yield or quality. Proof-of-concept studies demonstrating the feasibility of allogeneic OEC grafts with short-term immunosuppression in animal models of spinal cord injury have already shown some promising results including axon regeneration and recovery of directed forepaw reaching (Li et al. 2016).

To address the knowledge gap regarding the characteristics of human OECs in situ and to corroborate expression of rodent OEC markers in human foetal OECs, we undertook an immunocytochemical analysis of the 11–19 pcw human foetal olfactory system.

## Materials and methods

This project is covered by the Human Developmental Biology Resource (HDBR) HTA tissue bank licence and ethical approval. Details of approval terms can be found at www.hdbr.org. The human foetal material was provided by the joint MRC (Grant #G0700089)/Wellcome Trust (Grant # GR082557) Human Developmental Biology Resource (www.hdbr.org). Olfactory tissue blocks from aborted human foetuses (Table 1) were obtained with maternal consent and fixed in 4 % paraformaldehyde overnight. Samples 1–5 were dehydrated, paraffin embedded and sectioned by microtome. Tissue sections were deparaffinised in histoclear, rehydrated in graded alcohol and antigen retrieval was performed (60 min in 90 °C Tris/EDTA with 0.05 % Tween pH9). Samples 6–10 were cryoprotected in sucrose, frozen in OCT in a slurry of isopentane and dry ice and then cryosectioned.

**Table 1:** Details of the human foetal samples and tissue processing methods

Sample	Age (pcw)	Method
1	11	Paraffin
2	12	Paraffin
3	12	Paraffin
4	14	Paraffin
5	17	Paraffin
6	12	Cryosection
7	12	Cryosection
8	12	Cryosection
9	17	Cryosection
10	19 (no bulbs)	Cryosection

*pcw* post conception weeks

All tissue sections were blocked in antibody diluent (2 % milk, 1 % BSA and 0.1 % triton X-100) for 1 h at room temperature prior to incubating in primary antibodies (Table 2) overnight at 4 °C. Sections were then washed and incubated with the appropriate secondary antibodies (Invitrogen Alexafluor series; goat anti-chicken 546; goat anti-rabbit 488; donkey anti-mouse 488; donkey anti-rabbit 546, donkey anti-goat 633; goat anti-mouse IgM 488; DAPI) at 1:500 dilution at room temperature in blocking buffer for 2 h. Immunolabelling by all antibodies was compared to positive controls consisting of adjacent areas of the foetal brain, face, optic and oculomotor nerves, and negative controls where the primary antibody was omitted or replaced with an isotype control. Adjacent sections were stained with hematoxylin and eosin. Immunofluorescent micrographs were generated using either a Leica TCS SP confocal microscope (×63 or ×40 objective) or for large area montages with a Zeiss AxioScan Z.1 slide scanner (×20 objective).

**Table 2:** Details of the primary antibodies

Primary antibody	Manufacturer details	Host, dilution factor	Number of tissue sections labelled
TUJ-1	Covance, MMS-435P-250	Mouse monoclonal IgG, 1/500	56
S100	Dako, Z0311	Rabbit polyclonal IgG, 1/750	117
SOX10	Santa cruz, SC-17342	Goat polyclonal IgG, 1/50	112
GAP-43	Millipore, AB5220	Rabbit polyclonal IgG, 1/250	30
Nestin	Abcam, ab22035	Mouse monoclonal IgG, 1/100	14
GFAP	Millipore, MAB360	Mouse monoclonal IgG, 1/500	14
GFAP	Dako, Z0334	Rabbit polyclonal IgG, 1/500	51
Vimentin	Millipore, AB5733	Chicken Polyclonal IgG, 1/250	28
P75	Sigma, N5408	Mouse monoclonal IgG, 1/250	42
O4	Sigma, O7139	Mouse monoclonal IgM, 1/100	28
PSA-NCAM	Millipore, MAB5324	Mouse monoclonal clone 2-2B IgM, 1/200	28
NCAM	Abcam, AB5032	Rabbit polyclonal IgG, 1/250	14
NPY	Abcam, AB30914	Rabbit polyclonal IgG, 1/250	14
Isotype control	Sigma, M5909	Mouse IgM 1/100	3

## Results

The structure and cellular morphology of the olfactory system in our foetal samples were well preserved by our tissue processing methods. The OB, olfactory nerves and mucosa were easily distinguished in tissue sections at all foetal ages (11–19 pcw) as shown by H&E (Supplemental Fig. 1). The ONL was observed at all ages as a thin highly cellular layer covering the surface of the OB that became thicker in depth towards the rostral and inferior surfaces. In agreement with the literature we observed poor lamination in the OB at 11 & 12 pcw, by 14 pcw lamination became more evident and was clearly observed at 17 pcw (Humphrey 1940; Chuah and Zheng 1992; Müller and O’Rahilly 2004). At 17 pcw glomeruli were observed but appeared poorly developed similar to that described by Chuah and Zheng (1987).

**Fig. 1:**
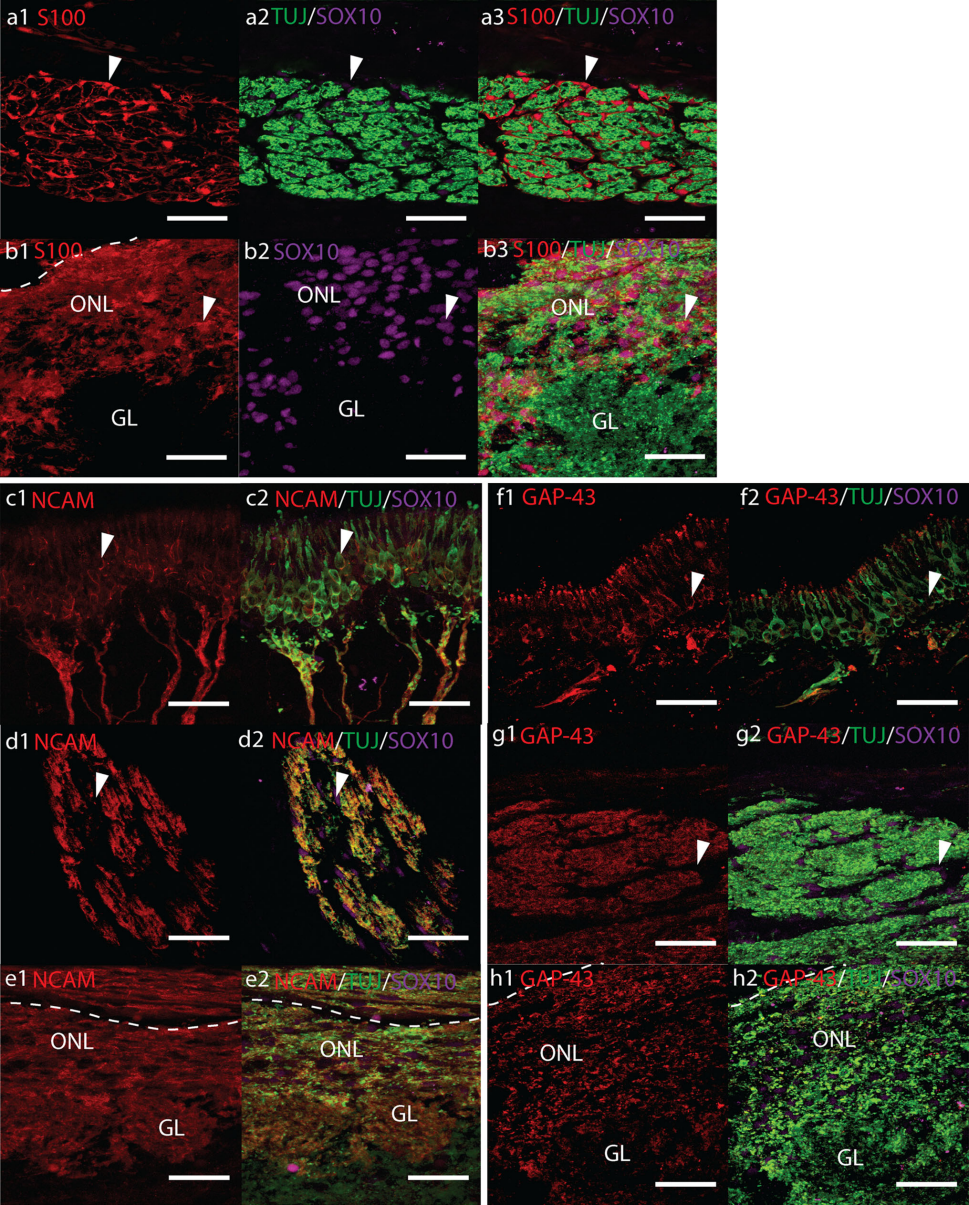
Confocal fluorescent micrographs showing sagittal cryosections of 17 pcw human foetal olfactory system immunolabelled with antibodies towards **a**, **b** TUJ (*green*), S100 (*red*) and SOX10 (*magenta*), **c**–**e** NCAM (*red*), TUJ (*green*), SOX10 (*magenta*) and **f**–**h** GAP-43 (*red*), TUJ (*green*), SOX10 (*magenta*). *Scale bar* 40 μm, *dashed white lines* indicate the surface of the olfactory bulb. **a** Precise cross-section of an olfactory nerve showing numerous SOX10/S100+ OECs (*white arrowheads*) surrounding bundles of TUJ+ olfactory axons. **b** SOX10/S100+ OECs (*white arrowheads*) are observed throughout the olfactory nerve layer (*ONL*) and surrounding but rarely within the glomerular layer (*GL*). **c**, **f** Olfactory receptor neurons (*white arrowheads*) and their axons co-labelled with TUJ and NCAM or GAP-43, respectively. **d**, **g** Cross-section of an olfactory nerve showing co-labelling of TUJ+ olfactory axons with NCAM and GAP-43. Co-labelling with SOX10 is not observed (*white arrows*). **e**, **h** Co-labelling of the *ONL* and *GL* with TUJ and NCAM or GAP-43, respectively

## Immunocytochemistry

### Immunolocalization of TUJ, S100, and SOX10

TUJ-1 and TUJ-20 antibodies detect beta III tubulin, a specific form of tubulin found in the microtubules of neurons. Both TUJ antibodies intensely labelled OSNs in the human foetal samples. At all developmental ages studied, SOX10+ nuclei and intense TUJ and S100 immunoreactivity was observed along the entirety of the olfactory nerves (ONs) and throughout the ONL (Fig. 1a–b and Supplemental Fig. 2a). The ONL surrounding the OB could be clearly identified by intense TUJ, S100 and SOX10 labelling. Double immunolabelling confirmed that S100+ cells were tightly associated with olfactory axons. Confocal micrographs of transverse sections of ONs clearly demonstrated SOX10 in the nucleus and S100 in the nucleus and cytoplasm of OECs that were ensheathing bundles of TUJ+ axons (Fig. 1a). Long S100 positive processes could be seen delineating axon fascicles in a tube-like fashion where ONs were cut in a longitudinal plane. The intensity of S100 labelling in the ONL surrounding the OB was less than that observed in the ONs, nonetheless, immunoreactivity in the ONL was still very strong. SOX10 was observed in the nuclei of S100+ OECs throughout the ONL (Fig. 1b). In the outer ONL, longitudinal S100+ OEC processes generally followed the orientation of the OB layers consistent with the morphology of ONs entering the ONL, whilst in the inner ONL, OECs appeared more heterogeneously orientated (Supplemental Fig. 2a). At 17 pcw, S100/SOX10+ OECs and their processes could be seen surrounding the outside but rarely within the developing TUJ+ glomeruli (Fig. 1b). S100 was not present within the olfactory epithelium (OE).

S100 Immunolabelling was also observed in chondrocytes in cartilage, weakly in the nucleus of cells in the deeper OB layers, and weakly in the cytoplasm of cells radiating outwards from the ventricle of the OB. SOX10 was also observed in the nucleus of duct cells in Bowman’s glands, and diffusely in presumptive sustentacular cells in the OE.

### Immunolocalization of NCAM and GAP-43

Immunoreactivity to TUJ, NCAM and GAP-43 was intense and tightly correlated in double labelling (TUJ/NCAM and TUJ/GAP-43) along the entire olfactory pathway (Fig. 1c–e and Supplemental Fig. 2b and Fig. 1f–h and Supplemental Fig. 2c, respectively). In the OE, the bipolar cell bodies of ORNs could be clearly identified by intense labelling with TUJ and slightly weaker to NCAM and Gap-43 (Fig. 1c, f, respectively). The OE generally possessed a pseudostratified columnar structure; however, in some areas the epithelium appeared thickened with numerous TUJ/GAP-43/NCAM+ cells of presumptive neuronal origin (Supplemental Fig. 2 b3). Within the nasal stroma, clusters of migrating TUJ/GAP-43/NCAM immunoreactive neurons were also observed.

In the ONs, triple labelling of cross-sections with SOX10/TUJ/NCAM or SOX10/TUJ/GAP43 showed tight overlap between TUJ and NCAM, and TUJ and GAP-43, but no clear overlap with SOX10 (Fig. 1d, g). Expression of NCAM and GAP-43 by OECs could not be clearly identified. The tight correlation of TUJ with NCAM and GAP-43 suggests these antigens were only present on OSN axons.

An intense band of NCAM and GAP-43 Immunolabelling was observed surrounding the circumference of the OB corresponding to the TUJ+ ONL (Fig. 1e, h and Supplemental Fig. 2 b, c). In 17 pcw samples, immunolabelling by NCAM and GAP-43 could also be observed in the developing TUJ+ glomeruli (Fig. 1e, h, respectively). Expression of NCAM and GAP-43 specifically on OECs in the ONL was difficult to confirm. Although tight overlap of NCAM and GAP-43 with TUJ was observed in the ONL suggesting neuronal origin, these antigens could still have been present on OEC processes in tight contact with axons not resolved by our micrographs.

TUJ, NCAM and Gap-43 immunoreactivity was also seen in small nerves surrounding arterioles and in facial peripheral nerves. Weak GAP-43 labelling was also observed in the meninges covering the brain (Supplemental Fig. 2c1). Occasionally, a layer of GAP-43 +/TUJ− immunoreactivity was observed underneath the OE (Supplemental Fig. 2d). Immunolabelling of adjacent sections showed this area was not reactive for S100, P75^NTR^, PSA-NCAM, nestin, NCAM, NPY or GFAP.

**Fig. 2:**
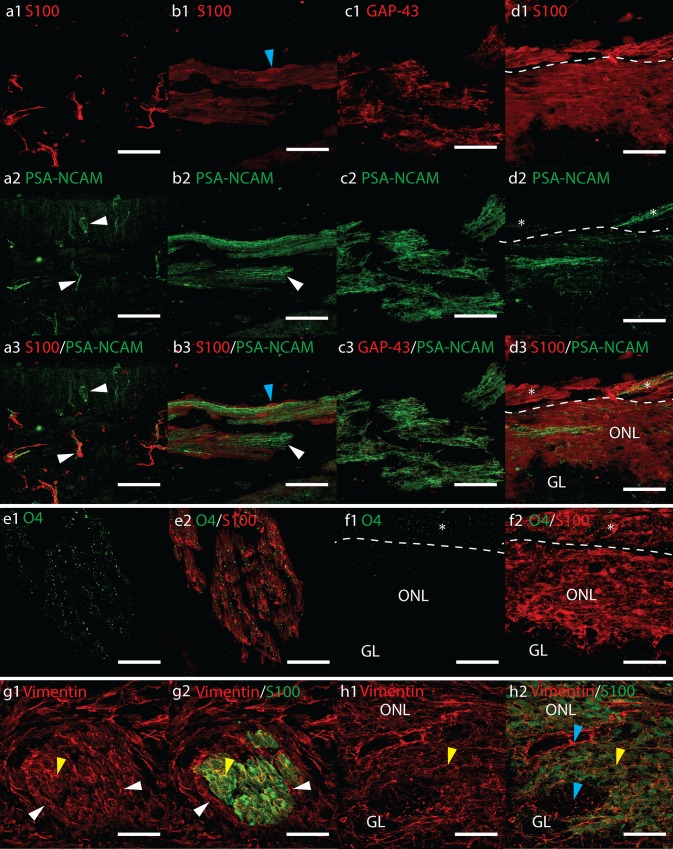
Confocal fluorescent micrographs showing sagittal cryosections of **a**–**d** 12 pcw and **e**–**h** 17 pcw human foetal olfactory system immunolabelled with antibodies towards **a**–**d** PSA-NCAM (*green*) and **a, b, d** S100 (*red*) or **c** GAP-43 (*red*), **e**–**f** O4 (*green*) and S100 (*red*), **g**, **h** vimentin (*red*) and S100 (*green*). All* scale bars* 40 μm. **d**, **f** *Asterisk* shows olfactory nerves merging with the olfactory nerve layer (*ONL*). **a** Olfactory epithelium showing PSA-NCAM immunolabelled olfactory receptor neurons and olfactory axons (*white arrowheads*). **b** Bundles of PSA-NCAM+ axons (*white arrowheads*) are ensheathed by S100+ OECs (*blue arrowheads*). **c** PSA-NCAM immunoreactivity in the olfactory nerves co-labels closely with GAP-43. **d** PSA-NCAM immunoreactivity in the ONL. **e** Strong O4 immunoreactivity overlapped with S100+ OECs in the olfactory nerves but not in the *ONL* (**f),** except where *ONs asterisk* merged with the *ONL*. **g, h** Co-labelling of vimentin with S100+ OECs (*yellow arrowheads*) is observed in **g** a cross-section of an olfactory nerve and in **h** the olfactory nerve layer. Olfactory nerve perineurial cells (*white arrowheads*) express vimentin but not S100. *Blue arrowheads* show vimentin+ S100− cells within the *ONL* and glomerular (GL)

### Immunolocalization of PSA-NCAM

PSA-NCAM labelling varied between the different foetal samples. In the 12 pcw foetuses PSA-NCAM labelled both intracranial and lamina propria ONs. Occasional PSA-NCAM+ ORNs were observed in the OE (Fig. 2a). PSA-NCAM immunoreactivity in the ONs possessed an axonal-like morphology that closely co-labelled with GAP-43 and was surrounded by S100 (Fig. 2b, c). This suggests the source of the PSA-NCAM was neuronal. Immunolabelling in the ONs was variable with some nerve bundles moderate to strongly immunoreactive and others very weakly immunoreactive. In some tissue sections individual nerve bundles could be followed from the OB to the OE. We observed entire bundles that were either very strong or not immunoreactive at all along the entire length of the nerve (Supplemental Fig. 3a). PSA-NCAM labelling in the ONL was variable and heterogeneous. Often it was either absent or appeared as very weak to moderately immunoreactive flecks. In the outer ONL long immunoreactive fibres were observed where PSA-NCAM+ nerve bundles entered the OB (Fig. 2d). PSA-NCAM immunolabelling was very similar between all the 12 pcw samples and the 19 pcw cryosectioned samples; however, the 17 pcw sample had no immunolabelling in the ONL and very rarely weak labelling in the ONs.

### Immunolocalization of O4

The majority of O4 immunolabelling was concentrated in the olfactory nerves; however, some background fluorescent speckles were occasionally seen deposited across the tissue sections. Omitting Triton-X100 from all buffers and antibody diluents did not prevent this.

We concluded that the decalcification, freezing and cryosectioning process could be responsible for dispersion of the O4 antigen. Despite the background speckles, it was possible to identify consistent immunolabelling in the olfactory nerves of all cryosectioned foetal samples where this immunolabelling was intense and clearly different from the background (Fig. 2e and Supplemental Fig. 3b). This same intense labelling was not observed in the ONL except where ONs merged with the bulb surface (Fig. 2f). O4 immunolabelling was not observed in background (no primary) or IgM isotype controls (Supplemental Fig. 3b1). It was not possible to isolate the origin of O4 to OECs or olfactory axons.

### Immunolocalization of Vimentin

Vimentin was intensely expressed in a wide range of cells within the olfactory system, in nervous tissue and in meninges (Supplemental Fig. 3c). Cross-section of the ONs showed co-labelling with S100+ positive OECs (Fig. 2g). Vimentin also labelled connective tissue and olfactory nerve perineurial cells surrounding the outside of OEC-axon units. Vimentin immunoreactivity was observed in cells in the basal region of the OE (Supplemental Fig. 3c). Strong vimentin expression was observed throughout the OB. In the inner and outer ONL, vimentin appeared to co-label with S100+ OECs; however, S100− vimentin+ cells were also observed, especially within the developing glomerular layer (Fig. 2h). Vimentin+ cells with weakly S100+ nuclei radiated outward from the bulb ventricle projecting from the deeper layers towards the more superficial layers in a similar manner to nestin (Supplemental Fig. 3c, d).

### Immunolocalization of P75^NTR^, S100, and SOX10

**Fig. 3:**
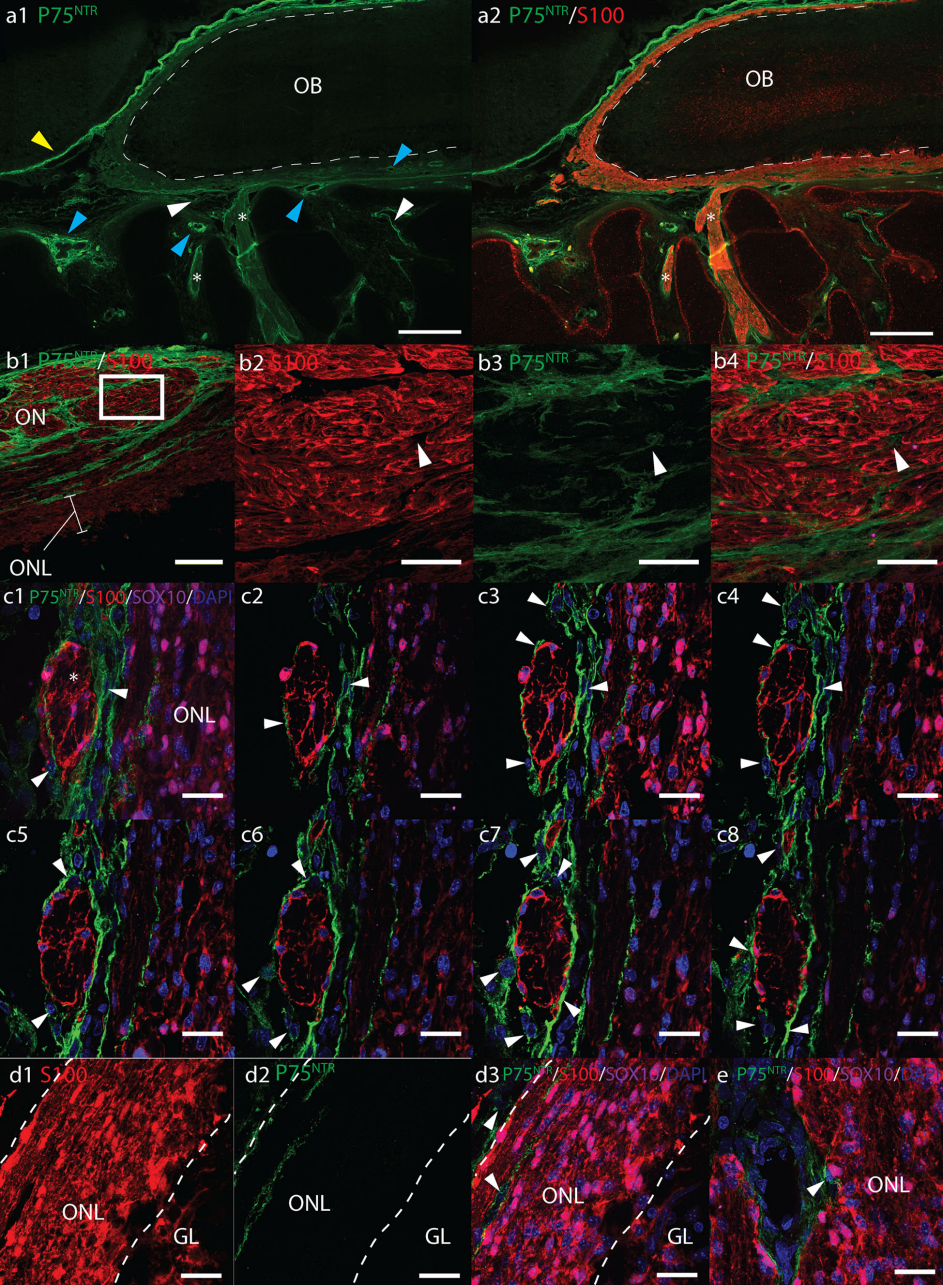
Fluorescent micrographs of sagittal cryosections from 17 pcw human foetal olfactory system immunolabelled with antibodies towards P75^NTR^ (*green*), S100 (*red*), SOX10 (*magenta*), and DAPI nuclear stain (*blue*). **a** Axioscan micrograph montages of the olfactory bulb (OB) and olfactory nerves (*ON*, *asterisk*). *Scale bar* 500 μm. S100 labels the entire olfactory nerve layer (*ONL*) and *ONs*, whereas P75^NTR^ surrounds the outside of the OB and ONs. P75^NTR^ labelling was also observed surrounding blood vessels (*blue arrowhead*); on cells within the nasal stroma (*white arrowheads*) and intensely expressed in meningeal tissue (*yellow arrowhead*). **b** Cross-section of olfactory nerves showing P75^NTR^+ processes (*white arrows*) surrounding S100+ OECs. **b1** *Scale bar* 100 μm. **b2**–**4** *Scale bar* 20 μm, shows *magnified images* of area within *white box* in **b1**. **c2**–**7** Serial optical sections through a 13 μm z-stack (**c1)** showing DAPI labelled nuclei and P75^NTR^+ processes of perineurial cells (*white arrows*) surrounding S100+ OECs within a cross-section of an olfactory nerve (*asterisk*). *Scale bar* 20 μm. **d1**–**3** In the outer *ONL*, P75^NTR^ is expressed on cells (*white arrowheads*) surrounding ONs as they enter the *ONL*, but not on SOX10/S100+ OECs. *Dashed white lines* show rough boundary of *ONL*, *scale bar* 20 μm. **e** P75^NTR^+ cells surround presumptive arterioles in the *ONL*,* scale bar* 20 μm

Along the entire olfactory pathway P75^NTR^+ cells delineated the outside of olfactory nerves (Fig. 3a–c). In transverse sections of ONs, P75^NTR^+ cells enwrapped bundles of S100/SOX10+ OEC-axon units (Fig. 3b1). Fine P75^NTR^+ processes were also observed between the OEC-axon units, but not within (Fig. 3b2–4 white arrows). In z-stack confocal micrographs of precise ON cross-sections, S100 and P75^NTR^ immunoreactivity appeared to occupy mutually exclusive areas with P75^NTR^ solely on processes of cells surrounding OECs, presumptive perineurial olfactory nerve fibroblasts (ONFs), but not expressed by OECs themselves (Fig. 3c). Rarely, individual fusiform P75^NTR^/SOX10/S100+ cells were observed free in the nasal stroma. No P75^NTR^ labelling was observed in the OE.

**Fig. 4:**
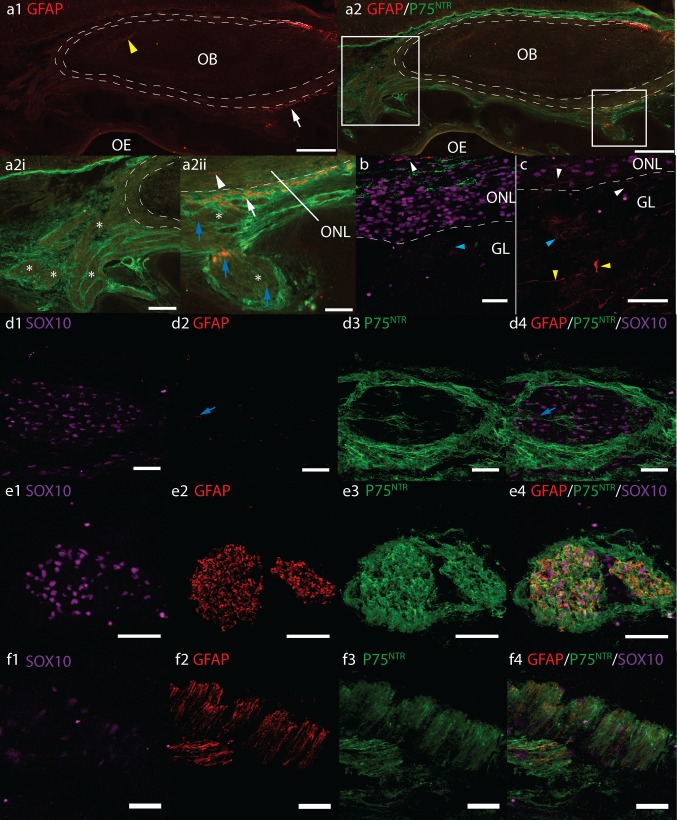
Axioscan fluorescent montages **(a)** and confocal fluorescent micrographs **(b**–**f)** of GFAP (*red*), P75^NTR^ (*green*) and SOX10 (*magenta*) immunoreactivity in sagittal cryosections of **a**–**d** 17 pcw and **f** 12 pcw human foetal olfactory system. *Scale bars* **a1, a2** 500 μm, **a2i** 200 μm, **a2ii** 100 μm, **b**–**e** 40 μm. **f** 20 μm. *Dashed white lines* roughly denote inner and outer boundaries of the olfactory nerve layer *ONL*, olfactory bulb *OB*, olfactory epithelium *OE*, glomerular layer *GL*, olfactory nerves *ON asterisk*. *Boxes* in **a2** show position of magnified areas in **a2i** and **a2ii**. **a** GFAP immunoreactivity in the *ONL* and *ONs* is sparse. *Yellow arrowhead* **a1** shows GFAP+ astrocytes, **a2i** shows the majority of *ONs asterisks* do not express significant GFAP, *white arrowhead* **a2ii** shows GFAP+ cells in *ONL*, *blue arrows* show sporadic GFAP in olfactory nerves *asterisk*. **b**, **c** GFAP in the *ONL* and *GL*, *white* and *blue arrowheads* show GFAP+ cells in *ONL* and glomerular layer, respectively. *Yellow arrowheads* show GFAP+ presumptive astrocytes in the deeper layers of the OB. **d** Sporadic GFAP in olfactory nerves which are surrounded by P75^NTR^+ perineurial cells. Co-labelling of GFAP & P75^NTR^ is not observed. Pattern of GFAP and P75^NTR^ co-labelling in peripheral facial nerves **e** is similar to that seen in occasional small nerve bundles in nasal stroma **f** and **a2ii** *white arrow*

Small blood vessels were often observed within the nasal mucosa and ONL surrounded by intensely P75^NTR^+ cells (Fig. 3a1, e, Supplemental Fig. 4a). P75^NTR^/SOX10/S100+ presumptive Schwann cells were often present in peripheral nerve bundles alongside these vessels (Supplemental Fig. 4a). Moderate to weak P75^NTR^ immunoreactivity was also present in numerous S100− cells dispersed within the nasal stroma (Fig. 3a1). These cells were more numerous intracranially surrounding ONs, situated along the intracranial surface of cribriform plate (Supplemental Fig. 4b) and within nerve foramen within the cribriform plate. P75^NTR^ also intensely labelled cells in the meninges surrounding the brain and OB (Fig. 3a1).

At the bulb surface, P75^NTR^ immunoreactivity was observed in cells surrounding S100+ OECs in ONs as they entered the outer ONL. Long P75^NTR^ positive streaks could be observed following the orientation of the OB layers on the surface of the OB and in the outer ONL, but rarely in the inner ONL (Fig. 3d). Where P75^NTR^ was observed within the inner ONL, this generally seemed to be associated with the presence of blood vessels (Fig. 3e). Triple immunolabelling demonstrated that S100/SOX10 and P75^NTR^ occupied mutually exclusive areas suggesting expression was not on OECs.

### Immunolocalization of GFAP and P75^NTR^

Anti-GFAP monoclonal and polyclonal antibodies resulted in differing patterns of immunolabelling. Monoclonal GFAP strongly labelled cells with an astrocyte morphology in specific areas of the foetal brain. However, immunoreactivity was not observed in any sections of the OB, ONs, lamina propria or OE, in any of the cryosectioned samples. Neither was immunoreactivity observed in sagittal sections of the developing eye, optic and oculomotor nerve. Immunoreactivity to the polyclonal GFAP in the brain was more widespread than the monoclonal GFAP. Additional areas of the forebrain were moderately immunoreactive. The optic nerve, oculomotor and facial nerves were strongly immunoreactive. In paraffin sections treated with antigen retrieval, strong GFAP immunoreactivity was also observed in the dura mater.

Generally polyclonal GFAP expression within the ONL was sparse (Fig. 4a, b). Occasionally elongated cells were visible orientated parallel to the OB layers or extending into the glomerular layer (Fig. 4a2ii, b, c white arrowheads). The frequency of these cells was variable and in some tissue sections immunolabelled cells were rarely observed. Co-labelling of GFAP with P75^NTR^ in the ONL was not apparent. Occasionally, weakly immunoreactive presumptive astrocytes were observed heterogeneously orientated within the developing glomerular (Fig. 4b, c, blue arrowheads). Many weakly GFAP+ presumptive astrocytes were also visible in the deeper layers of the bulb, the majority of which were orientated parallel to the olfactory bulb layers, with some projecting perpendicular towards the developing glomerular layer (Fig. 4a1, c yellow arrowheads).

In general, polyclonal GFAP immunoreactivity was absent from the ONs apart from occasional sporadic immunoreactive streaks within the nerve bundles (Fig. 4a2ii, d blue arrows). GFAP immunolabelling did not appear to co-label with P75^NTR^ cells surrounding the ONs (Fig. 4d). Occasionally, strong GFAP immunoreactivity was observed throughout small nerve bundles (Fig. 4a white arrows, f). The pattern of GFAP/P75^NTR^ co-labelling in these nerves was comparable to that observed in peripheral facial nerves (Fig. 4e) and those surrounding blood vessels suggesting these nerve bundles may not have been olfactory in origin. Similar GFAP and P75^NTR^ co-labelling was not observed in nerves that were obviously olfactory in origin i.e. large nerve bundles clearly entering the olfactory bulb (Fig. 4a2i).

### Immunolocalization of nestin

**Fig. 5:**
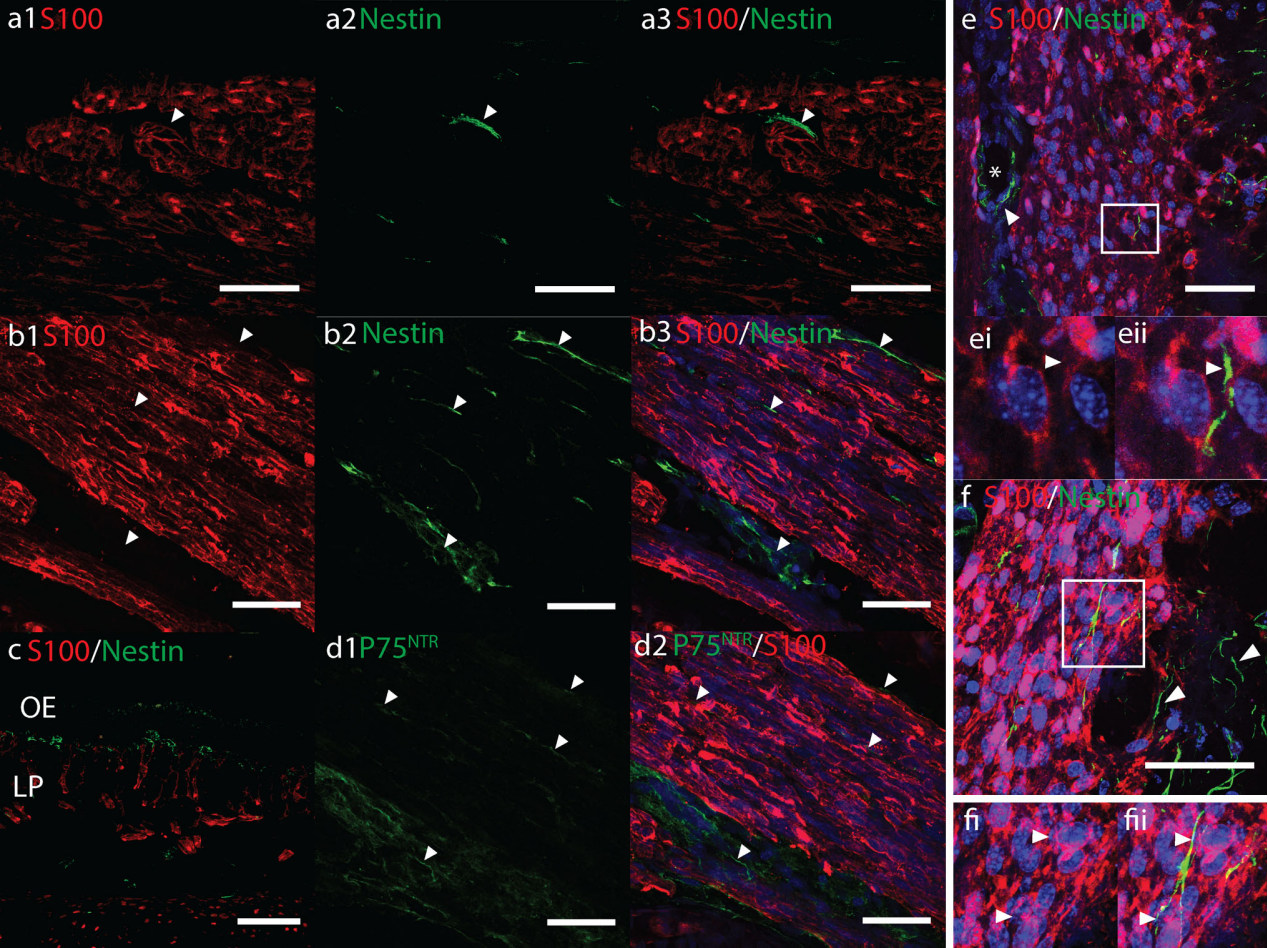
Confocal fluorescent micrographs showing **a**–**c, e, f** nestin (*green*) and **d** P75NTR (*green*), **a**–**f** S100 (*red*) immunoreactivity in sagittal cryosections of 17 pcw human foetal olfactory system. *Scale bars* 40 μm. **a** *White arrowheads* show nestin immunoreactivity surrounding the outside of S100+ OECs in cross-sections of olfactory nerves (*ON*). **b** Nestin immunoreactivity surrounding *ONs* and between OEC-axon units (*white arrowheads*) in longitudinal sections of *ONs* appears similar to P75NTR immunoreactivity **d** in adjacent tissue sections**. c** Nestin immunoreactivity in the olfactory epithelium (*OE*) and lamina propria (*LP*). **e**, **f** *White boxes* show magnified areas of nestin+ S100− cells (*white arrows*) in the *ONL*. **e** also shows nestin+ cells around a blood vessel *asterisk* in the *ONL*

In precise cross-sections of ONs, nestin immunoreactivity was not observed in S100+ OECs (Fig. 5a). In a similar manner to P75^NTR^ immunolabelling (Fig. 5d), nestin+ cells were occasionally observed in-between and surrounding the outside of bundles of S100+ OEC-axon units (Fig. 5a, b, white arrows). Nestin immunoreactivity was also often present in the lamina propria and OE, but not co-labelling with S100+ OECs (Fig. 5c). Nestin immunoreactivity was most obvious within the OB. Numerous intensely labelled cells radiated from the bulb centre towards the periphery. Some co-labelled with moderate to weak nuclear and cytoplasmic S100 (Supplemental Fig. 3d). These cells reduced in numbers towards the superficial layers of the OB; in the ONL comparatively few nestin immunoreactive cells were observed. These sporadic cells spanned both the inner and outer ONLs and were heterogeneously orientated with some following the direction of the OB layers. Co-labelling with S100/SOX10 OECs in the ONL was not obvious (Fig. 5e, f). Often S100− nestin+ cells were present around blood vessels, around the outside of nerve bundles entering the ONL, and were common in the glomerular layer (Fig. 5e, f, Supplemental Fig. 3d). Nestin immunoreactivity was intense in the muscles of the eye but was not observed co-labelling with S100+ oculomotor nerves.

## Discussion

The immunocytochemical characterisation of OECs in the olfactory system of animal models such as rats and dogs has enhanced our understanding of OEC physiology and established a range of antigenic markers that can be used to identify OECs in experimental cultures. Identification of surface markers such as P75^NTR^ has also enabled the development of important purification techniques to enhance culture yields and eliminate unwanted cell types. In contrast, limited information is available regarding the antigenic characteristics of OECs in situ in the adult and developing human olfactory bulb and olfactory nerves. Studies in various animal models have shown slight but important differences in the characteristics of OECs between species. Demonstrating the identity and purity of cell transplants is a prerequisite to gaining authorisation for clinical trials of advanced therapeutic medicinal products. Thus, a thorough characterisation of human OECs in situ would be highly beneficial for the clinical development of OEC transplants. To address this, we investigated the in situ expression of common rodent OEC markers in the developing 11-19 pcw human foetal olfactory system. We assessed immunoreactivity to S100, P75^NTR^, PSA-NCAM, Gap-43, vimentin, nestin, NPY, GFAP, and O4, and to a lesser-known marker for OECs: SOX10.

Our results indicate that the antigenic properties of OECs in the developing human have many similarities but also some important differences with rodent OECs in situ. Franceschini and Barnett (1996) provide a detailed description of the antigenic characteristics of the developing and postnatal rat olfactory system (Franceschini and Barnett 1996). From E14-P0, the olfactory nerves were described as consistently PSA-NCAM+ on the inside surrounded by a thin filamentous P75^NTR^+ outer layer. At the ventral surface of the OB, PSA-NCAM expressing cells extended through openings in a P75^NTR^+ layer that wrapped around the PSA-NCAM+ tissue. PSA-NCAM immunoreactivity was restricted to the outer ONL until birth when it was also detected at the ONL/GL boundary. This configuration of an external P75^NTR^ layer surrounding a PSA-NCAM+ layer was observed along the entire olfactory pathway. They concluded that the PSA-NCAM+ tissue represented astrocyte-like OECs enwrapped by the P75^NTR^+ Schwann-like OECs. An additional layer of P75^NTR^ immunoreactivity covering the OB and entire cortex was also observed and identified as meningeal tissue. The migratory mass and olfactory axons extending from the OE to the OB consistently labelled with NCAM, vimentin and GAP-43 throughout development.

Our results showed some similarities with the configurations of immunoreactivity described by Franceschini and Barnett (1996). We observed consistent immunolabelling with vimentin, S100, NCAM and GAP-43 along the entire length of the ONs from the OE to the OB in our human foetal samples. Furthermore, we also observed P75^NTR^ in a meningeal layer covering the OB, and in similar configurations with PSA-NCAM immunolabelling. Consecutive double-labelled sections revealed that PSA-NCAM+ tissue occupied areas surrounded by P75^NTR^+ tissue in the ONs, similar to that described by Franceschini and Barnett. However, our results suggest a different cellular origin of these two antigens in the developing human foetus from that proposed by Franceschini and Barnett. Our high magnification confocal fluorescent micrographs of the olfactory nerves revealed three layers: S100− vimentin+ P75^NTR^+ ONFs surrounding S100+, SOX10+ and vimentin+ OECs which ensheathed NCAM, GAP-43, TUJ and PSA-NCAM+ bundles of ORNs axons.

We identified OECs by robust S100 and SOX10 immunolabelling that was consistently observed in cells with the morphology of OECs that tightly ensheathed TUJ+ ORN axons in olfactory nerves, at all foetal ages studied. S100/SOX10 expression in OECs was consistent along the entire olfactory pathway and throughout the ONL and was thus used where possible in triple antibody labelled sections as a marker for OECs. We also confirmed NCAM and GAP-43 as markers of ORN axons and not OECs in olfactory nerves by co-labelling with TUJ (ORNs axons) and SOX10 (OECs). Our results were consistent with previous studies showing NCAM and GAP-43 in ORN axons in human and mouse (Chuah and Au 1992; Au et al. 2002; Iwema and Schwob 2003; Maresh et al. 2008). Co-labelling with these markers revealed that PSA-NCAM was present in ORN axons but not obviously in OECs. It is possible that PSA-NCAM was present on OEC surfaces in contact with ORN axons but this was not distinguishable by fluorescence microscopy. Nevertheless, the pattern of PSA-NCAM immunoreactivity we observed was consistent with previous reports that only subpopulations of immature ORNs express this antigen (Miragall and Dermietzel 1992).

Our most significant finding was that human OECs do not appear to express P75^NTR^ but perineural ONFs surrounding OECs in olfactory nerve fascicles do strongly express P75^NTR^. This was consistent among all the samples analysed and throughout the olfactory system in both the ONs and outer ONL. Expression of P75^NTR^ by ONFs and not OECs was most obvious in precise cross-sections and z-stack confocal images of olfactory nerves. Co-labelling with S100 and SOX10 demonstrated that P75^NTR^ was on cells surrounding OECs and did not appear to be on OECs themselves. Without co-labelling with S100, the similar distribution and morphology of P75^NTR^ immunolabelling in the olfactory nerves could be mistaken for labelling in OECs. These results are similar to those found in adult canine, guinea pig and cat, but in contrast to rat and murine OECs (Gong et al. 1994; Franceschini and Barnett 1996; Au et al. 2002; Au and Roskams 2003; Bock et al. 2007; Smithson and Kawaja 2009). These results are also in contrast with Liu et al. (2010) who observed substantial P75^NTR^ immunoreactivity in the ONL and surrounding glomeruli in the olfactory bulb in 20 pcw human foetuses (Liu et al. 2010). Differences between our study and Liu et al. could be due to differing antibodies or foetal sample age. Strong immunostaining in peripheral nerves in control tissue sections taken from the same foetal samples, and in the meningeal tissue (which is well reported) provides confidence that the P75^NTR^ antibody used in this study was effective.

Electron micrograph studies have shown the configuration of OEC ensheathment of olfactory axons and their compartmentalisation into fascicles by basal lamina and ONFs differs among species (Herrera et al. 2005; Kawaja et al. 2009; Smithson and Kawaja 2009). In rodents, spaces between OEC-axon units are packed with collagen fibres and only a single loose layer of ONFs surrounds the outside of ONs (Field et al. 2003). In larger mammals such as cats and non-human primates *Macaca fascicularis*, each OEC-axon unit within a larger fascicle is separated by collagen fibres and a thin single cell layer endoneurium-like fibroblastic process lacking a basal lamina (Herrera et al. 2005). Multiple fascicles are then surrounded by a second layer of perineurial ONFs. Our results suggest the configuration in humans is similar to *Macaca fascicularis*. The P75^NTR^+ layers we observed surrounding the S100/SOX10+ OEC-axon units and fascicles are these endoneurial and perineurial ONFs. Smithson et al. (2009) and Bock et al. (2007) reported a similar configuration of P75^NTR^+ cells surrounding olfactory nerves in adult guinea pigs, cats and dogs and concluded these cells were ONFs. They did not observe P75^NTR^ in OECs in these species (Bock et al. 2007; Smithson and Kawaja 2009).

Our tissue sections of the foetal olfactory system often contained nerves of peripheral origin coursing through the nasal mucosa and alongside blood vessels in both the peripheral and intracranial compartments of the olfactory system. These small vessels were present within the outer and inner ONL and along the surface of the olfactory bulb. Similar to olfactory nerves we observed P75^NTR^ immunolabelling in perineurial fibroblasts surrounding peripheral nerves; however, in contrast to OECs, Schwann cells in these nerves also strongly expressed P75^NTR^ determined by clear overlap with S100 immunoreactivity. Schwann cells also expressed significantly more GFAP compared to OECs. Similar to that described in the developing rodent olfactory system, GFAP expression in OECs in the olfactory nerves and ONL was very sparse whilst in peripheral nerves, GFAP was intense and abundant throughout the entire nerve bundle. These data show clear differences in the phenotype of Schwann cells and OECs during the developmental ages studied.

The finding that P75^NTR^ is expressed by Schwann cells and ONFs but not by OECs in situ has implications for developing techniques to purify, identify and culture human OECs. Traditional methods developed in rodents whereby OECs are isolated by P75^NTR^ immunopanning the initial cell suspension would result in a culture that did not contain OECs, only Schwann cells and ONFs. Alternatively, P75^NTR^ immunopanning after a period in culture would allow time for OECs to upregulate P75^NTR^, but would also isolate Schwann cells and presumably ONFs. Using both these protocols sequentially to first remove Schwann cells and ONFs, and then after a culture period to isolate OECs could provide highly pure OEC cultures similar to protocols described for canine OECs (Bock et al. 2007).

Transplants of OECs generated from the digestion of whole human foetal olfactory bulbs are already being used to treat hundreds of patients for various ailments including SCI, stroke, cerebral palsy and neurodegenerative diseases such as ALS (Huang et al. 2006, 2009; Chen et al. 2012, 2013, 2014; Xi et al. 2013). The transplanted cells in these studies have been described as OECs and characterised using antibodies towards P75^NTR^. No selective purification methods have been described to isolate OECs from other cells of the olfactory bulb indicating these cultures are likely to contain a wide range of cells and not just OECs. Liu et al. (2010) characterised human foetal OB cultures generated using similar methods and found them to contain just 23 % P75^NTR^+ cells. Other cells in the cultures were GFAP+, S100+ and nestin+, possibly neural stem and precursor cells, reactive astrocytes or microglia (Liu et al. 2010). Another report by Guest et al. (2006) found similar cultures described as OECs contained nestin and GFAP but no S100 and were morphologically reminiscent of human neurosphere cultures; the transplants were not clearly OECs (Guest et al. 2006). Outcomes from these human foetal OEC clinical studies have been controversial. The ability to correctly identify and purify human OECs is vital to understanding and developing effective OEC therapies. We did not observed nestin in OECs in the ONL of our foetal OB cryosections however, many other cells that were not OECs in the glomerular and deeper OB layers did express nestin, suggesting this marker is not useful for identifying human OECs generated from OB tissue.

SOX10 is a transcription factor required for the differentiation of Schwann cells and for the development of OECs in mice and chick (Kelsh 2006; Barraud et al. 2010; Forni and Wray 2012; Pingault et al. 2013). Elucidating the developmental pathways of OECs will be essential for developing differentiation protocols to produce stem cell derived human OECs. SOX10 has also been observed in OECs in the mucosal olfactory nerves of a single 8-week human foetus (Pingault et al. 2013). Our observations of SOX10 confirm the presence of this key transcription factor in OECs throughout the ONs and ONL in the 11–19 pcw developing human.

Often, OECs are described as several different populations in vivo depending on their expression of various markers. Franceschini and Barnett (1996) described two OEC populations in rodent: astrocyte-like expressing PSA-NCAM and fibrous GFAP, and Schwann-like expressing P75^NTR^ with diffuse GFAP (Franceschini and Barnett 1996). In the olfactory bulb of mice, Au et al. (2002) identified P75^NTR^− S100+ NPY+ OECs in the inner ONL and P75^NTR^/PSA-NCAM+ OECs in outer ONL (Au et al. 2002). Pixley (1992) also described Schwann-like (S100/P75^NTR^/GFAP+) and astrocyte-like (GFAP+ and S100/P75^NTR^−) OECs in situ in the olfactory nerves of neonatal rats. Intensely GFAP+ astrocyte-like OECs occurred as isolated cells within the ONs, orientated parallel with the longitudinal axis of the nerve (Pixley 1992). We observed one population of OECs within both the ONs and ONL of the developing human olfactory system that expressed S100, SOX10, vimentin and heterogeneously small amounts of fibrous GFAP (Table 3). We were unable to confirm S100 co-labelling in GFAP+ OECs as both antibodies were raised in rabbit and our anti-mouse GFAP only detected GFAP in small populations of astrocytes in the forebrain. The GFAP+ OECs we observed were similar to that described by Pixley (1992) occurring as isolated cells orientated parallel with the nerve (Fig. 4d). These cells may represent a second OEC population, the astrocyte-like OECs. We also describe another cell type that we assumed to be ONFs based on its anatomical morphology i.e. ensheathing S100+ OECs and not TUJ ORN axons. This cell type was S100− vimentin+ GFAP− P75^NTR^+, some of which also expressed nestin.

**Table 3:** Summary of the immunohistochemical analysis of the human foetal olfactory system

**Location**	**Gap-43**	TUJ	**S100**	**GFAP** **mc**	**GFAP** **poly**	**P75** ^**NTR**^	**Sox10**	**PSA-NCAM**	**NCAM**	**Vimentin**	**Nestin**	**O4**	NPY
Olfactory nerve	+++§§§	+++§§§	+++§§§	A	++ ± to §	+++§§§	+++§§§	A to +++ §§§	+++§§§	+++§§§	+§	++§§	A
Olfactory nerve layer (outer/inner 17 pcw only)	+++§§§	+++§§§	+++§§§	A	+§	+++ § (+++§/+++±)	+++§§§	A to ++ §	+++§§§	+++§§§	++§	A (+ ±/A)	A
Glomerular layer (17 pcw only)	++§§§	+++§§§	A	A	+§	A	A	A	+++§§§	++§§	++§§	A	A
Olfactory epithelia	+§§	+++§§§	++ ±	A	A	A	+§§	++ §	+++§§§	++§§	A	A	A
Co-label with p75?	ND	No	In peripheral nerves.	ND	In peripheral nerves.	NA	Yes - In Schwann cells and rarely in olfactory perineural cells.	ND	ND	ND-but labelling of adjacent tissue sections suggests yes.	ND-but labelling of adjacent tissue sections suggests yes.	ND	ND
Clearly expressed by OECs?	No	No	Yes, extensively.	No	Yes, in small amounts.	No	Yes, extensively.	No	No	Yes, extensively.	No	ONL: No ON:Unsure	No

Strength of IR: *A* Absent, + weak, ++ strong, +++ very strong, frequency of IR: rarely positive cells ±, sparse §, often and consistent §§, abundant and consistent §§§. *ND* not done, *ONL* olfactory nerve layer, *ON* olfactory nerve

We did not observe any differences in the immunolabelling characteristics of OECs between the ONs and ONL other than O4, which labelled ONs but was absent in the ONL. O4 was previously considered to be a surface marker for rodent OECs and has been used by several groups to enrich OEC cultures using FACs (Franceschini and Barnett 1996). O4 immunoreactivity on rodent OECs in vivo was initially described by immunofluorescence. Later studies utilising immunoelectron microscopy revealed this antigen was actually present on ORN axons and not OECs (Wewetzer et al. 2005). This study concluded that O4 observed on OECs in vitro was from phagocytosed axonal fragments and not endogenous expression; this has been disputed and remains unresolved. We were unable to identify the origin of O4 immunoreactivity in the human foetal olfactory system and thus, this requires further investigation by immunoelectron microscopy. We were also unable to identify a NPY+ OEC population equivalent to that described by Ubink in the ONL of embryonic rats suggesting this neurotrophic factor may not be involved in human olfactory development at the foetal ages studied (Ubink and Hökfelt 2000).

### Antigenic properties of ONFs

The identification of P75^NTR^+ S100− ONFs in this study raises a number of important questions: Do we see similar P75^NTR^ immunoreactivity in adult human ONFs, alluded to by Holbrook et al. 2016? What are the morphological and antigenic characteristics of these cells in culture? Do they continue to express P75^NTR^ in vitro and if so, what proportion of these cells persist in OEC cultures that are traditionally characterised based on P75^NTR^ purity. In rodent OEC cultures, ONFs are normally characterised by positive labelling for fibronectin, Thy-1, Smooth muscle actin and negative for P75^NTR^ (Li et al. 2008; Toft et al. 2013). This is similar to the phenotype of endoneurial fibroblasts found in the peripheral nerves of mice that along with Schwann cells differentiate from a non-neuronal restricted progenitor cell originating from the neural crest (Joseph et al. 2004). This non-neuronal restricted progenitor cell is characterised by expression of P75^NTR^, S100b and desert hedgehog. Differentiation along the fibroblast lineage results in the loss of P75^NTR^ and desert hedgehog and expression of Thy-1. Human ONFs clearly differ antigenically from rodent ONFs and peripheral nerve endoneurial fibroblasts. Dissecting the function of P75^NTR^ expression is difficult as this pan-neurotrophin receptor and NOGO-66 coreceptor mediates a wide range of extremely complex signals, many of which are still being deciphered. P75^NTR^ is expressed in neurons and glial cells as well as many mature non-neuronal cells such as endothelial cells and perivascular fibroblasts. It is also expressed in various types of mesenchymal cells and embryonic and adult stem cells. Among many functions, P75^NTR^ mediates cell death and survival, proliferation, differentiation, migration, and has essential roles in controlling the development of the nervous system including neuronal survival, neurite outgrowth and retraction, and myelination (Rabizadeh and Bredesen 2003). Schwann cell P75^NTR^ is vital for promoting peripheral nerve regeneration after injury (Zhou and Li 2007). P75^NTR^ is also involved in controlling pluripotency and modulating cell-fate decisions in stem cells (Tomellini et al. 2014). The significance of P75^NTR^ in human ONFs remains to be investigated.

As well as P75^NTR^, our results indicate that a proportion of human ONFs also express nestin. Nestin is a cytoskeletal intermediate filament protein associated with an immature phenotype expressed abundantly in progenitor and multipotent stem cells and in various tissues during development, particularly in the nervous system (Michalczyk and Ziman 2005). During differentiation nestin is normally replaced by cell type specific intermediate filament proteins but can be re-expressed transiently during tissue regeneration and wound healing. The expression of nestin in human ONFs is not entirely unexpected as nestin has been previously reported in myofibroblasts in various different tissues (Kishaba et al. 2010), in fibroblasts cultured from human olfactory mucosa biopsies (Wu et al. 2013), and in a sub-population of endoneurial fibroblast-like cells in rodent and human peripheral nerves (Richard et al. 2014). Richard et al. (2014) reported that after peripheral nerve injury in rodents, the number of nestin expressing endoneurial fibroblast-like cells significantly increased leading them to speculate that these cells may have progenitor-like properties. Similarly, the presence of nestin in human ONFs could indicate progenitor-like properties. It is currently unknown to what extent human ONFs are needed in OEC transplants to achieve therapeutic outcomes. Previous studies in rodents have suggested that ONFs are a crucial component of therapeutic OEC transplants facilitating the formation of OEC bridges through which axons can regenerate across growth inhibitory CNS tissue. Electron microscopy studies have shown that after transplantation into spinal cord lesions, ONFs wrap around OEC channels forming a sheath similar to their physiology in situ (Li et al. 1998 *).* During culture, ONFs also contribute to the production of a gel-like endogenous matrix that provides a useful vehicle for transplanting OECs to the injury site. Thus, although P75^NTR^ immunopanning as described may allow the generation of human OECs cultures free from Schwann cells, further surface markers will be needed to isolate ONFs from Schwann cells to investigate their role in the regenerative properties of OEC transplants.

In summary, we show that human foetal OECs possess both similarities and important differences with rodent OECs in situ. Most significant of these findings is that ONFs and not OECs express P75^NTR^ in the developing foetus. However, this antigen could still be used in protocols to remove P75^NTR^+ Schwann cells and fibroblasts and eventually isolate OECs after P75^NTR^ upregulation in vitro, to generate purified cultures from foetal tissue. We define OECs throughout the 11–19 pcw human olfactory system as S100/vimentin/SOX10+ with very small amounts of GFAP. Expression of O4 on OECs and not ORN axons in situ remains to be proven and presently it is not a robust surface marker for OEC purification. PSA-NCAM is also not a suitable surface marker for OEC purification owing to its heterogeneous expression on ORN axons and lack of reliable labelling of OECs in the human foetus. Our results highlight the importance of corroborating cell markers when translating cell therapies from animal models to the clinic.

## Electronic supplementary material

Below is the link to the electronic supplementary material.

**Supplemental Fig.** **1** Haematoxylin & Eosin histochemistry of **a** 12pcw and **b** 17 pcw foetal olfactory system. The olfactory nerve layer (ONL) can be observed as a thin but highly cellular layer surrounding the circumference of the olfactory bulb (OB). Scale bars: **a1** & **b1** 500 μm, **a2** & **b2** 100 μm. CP cribriform plate, OE olfactory epithelium, ON olfactory nerve, GL glomerular layer. Dashed lines in **a2** show outline of the ONL, * shows developing glomeruli. (TIFF 13192 kb)

**Supplemental Fig.** **2** Axioscan fluorescent montages (**a-c**) and confocal fluorescent micrographs (**d)** showing sagittal cryosections of 17 pcw human foetal olfactory system immunolabelled with antibodies towards TUJ (green), SOX10 (magenta) and **a** S100 (red), **b** NCAM (red), **c** & **d** GAP-43 (red). Scale bar **a-c** 500 μm **d** 40 μm, CP cribriform plate, LP lamia propria, OE olfactory epithelium, OB olfactory bulb, ONL olfactory nerve layer, ON olfactory nerve. White arrows in **c1** and **d** show weak GAP-43 expression in meninges and under the olfactory epithelium, respectively. (TIFF 29333 kb)

**Supplemental Fig.** **3** Axioscan fluorescent montages showing sagittal cryosections of **a** 12 pcw and **b-d** 17 pcw human foetal olfactory system immunolabelled with antibodies towards **a** PSA-NCAM (green) & GAP-43 (red), **b1** O4 IgM isotype control, **b2-b4** O4 (green) & GAP-43 (red), **c** S100 (green) & vimentin (red), **d** nestin (green) & S100 (red). Scale bar 500 μm, CP cribriform plate, OE olfactory epithelium, OB olfactory bulb. In **a** the olfactory nerve layer (*) has peeled away from the OB and adhered to the CP; white arrowheads point to bundles of olfactory nerves showing heterogeneous labelling with PSA-NCAM. **c** white arrowhead points to vimentin+ meninges, white arrow indicates vimentin in the OE, blue arrowhead shows vimentin/S100+ cells radiating outward from the OB centre. **d** blue arrowheads point to nestin/S100+ cells radiating outward from the OB centre. (TIFF 30784 kb)

**Supplemental Fig.** **4** Confocal fluorescent micrographs of sagittal cryosections of 17 pcw human foetal olfactory system immunolabelled with antibodies towards P75^NTR^ (green), S100 (red) and DAPI (blue). **a** arteriole (white arrow) in the vicinity of olfactory nerves surrounded by large and small peripheral nerve bundles (yellow arrowheads), scale bar 100 μm. **b** P75^NTR^+ S100− cells (white arrows) surrounding the outside and covering the surface of the olfactory bulb, scale bar 40 μm. (TIFF 3491 kb)

## Abbreviations

EPL: External plexiform layer
GFAP: Glial fibrillary acid protein
GL: Glomeruli layer
IPL: Internal plexiform layer
MCL: Mitral cell layer
NCAM: Neural cell adhesion molecule
NPY: Neuropeptide Y
OB: Olfactory bulb
OEC: Olfactory ensheathing cell
ONF: Olfactory nerve fibroblast
ON: Olfactory nerve
ONL: Olfactory nerve layer
ORNs: Olfactory receptor neurons
P75^NTR^: Low affinity neurotrophin receptor
Pcw: Post conception weeks
PSA-NCAM: Polysialylated neural cell adhesion molecule
