# Supplementary figures and images for: Common olfactory ensheathing glial markers in the developing human olfactory system
Source: Brain Struct Funct. 2016 Oct 7;222(4):1877–95. doi: 10.1007/s00429-016-1313-y (PMC5406434; doi:10.1007/s00429-016-1313-y)

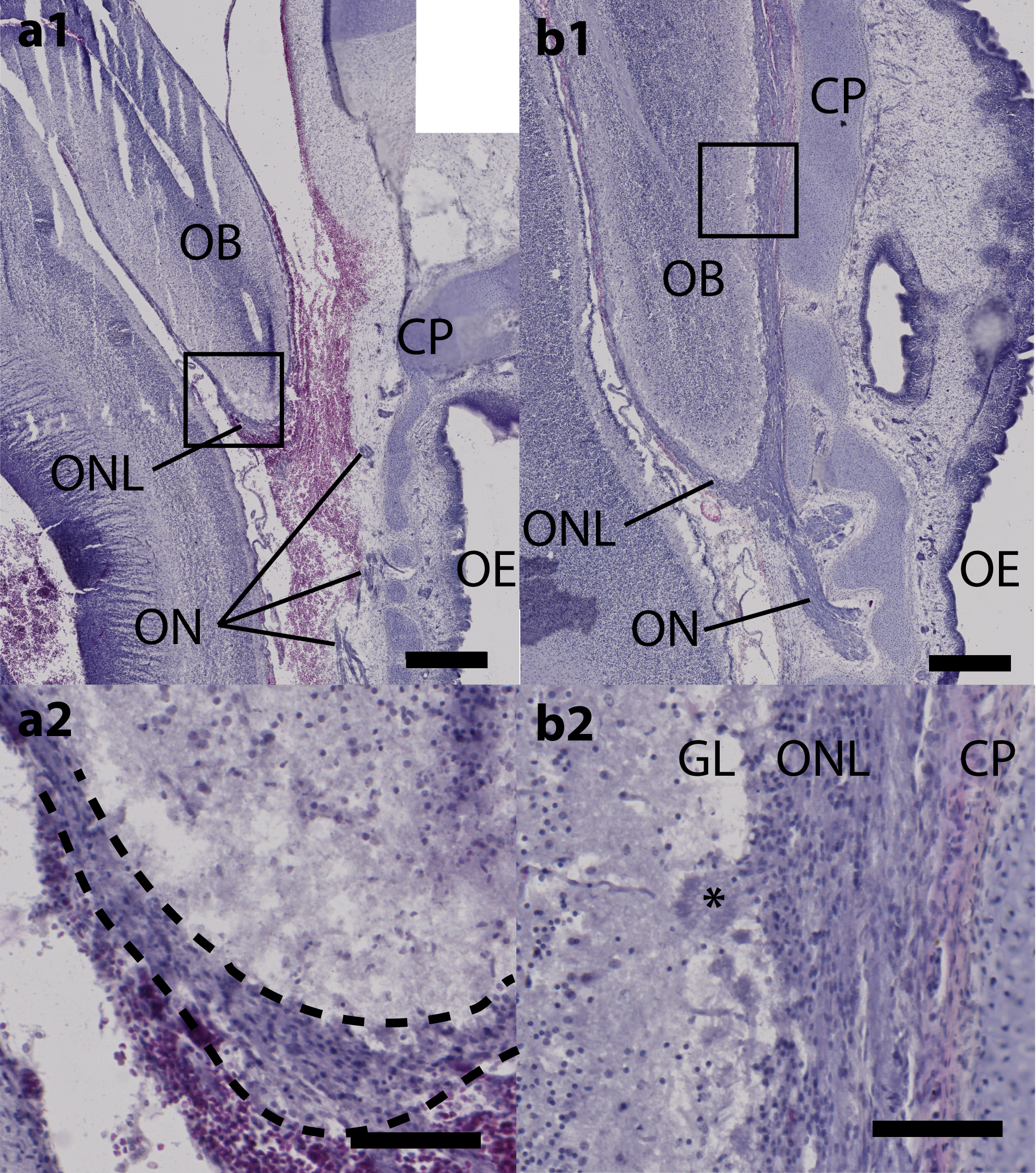

Supplement: Supplementary file 1 — Supplemental Fig. 1 Haematoxylin & Eosin histochemistry of a 12pcw and b 17 pcw foetal olfactory system. The olfactory nerve layer (ONL) can be observed as a thin but highly cellular layer surrounding the circumference of the olfactory bulb (OB). Scale bars: a1 & b1 500 μm, a2 & b2 100 μm. CP cribriform plate, OE olfactory epithelium, ON olfactory nerve, GL glomerular layer. Dashed lines in a2 show outline of the ONL, * shows developing glomeruli. (TIFF 13192 kb) [file 429_2016_1313_MOESM1_ESM.tif]

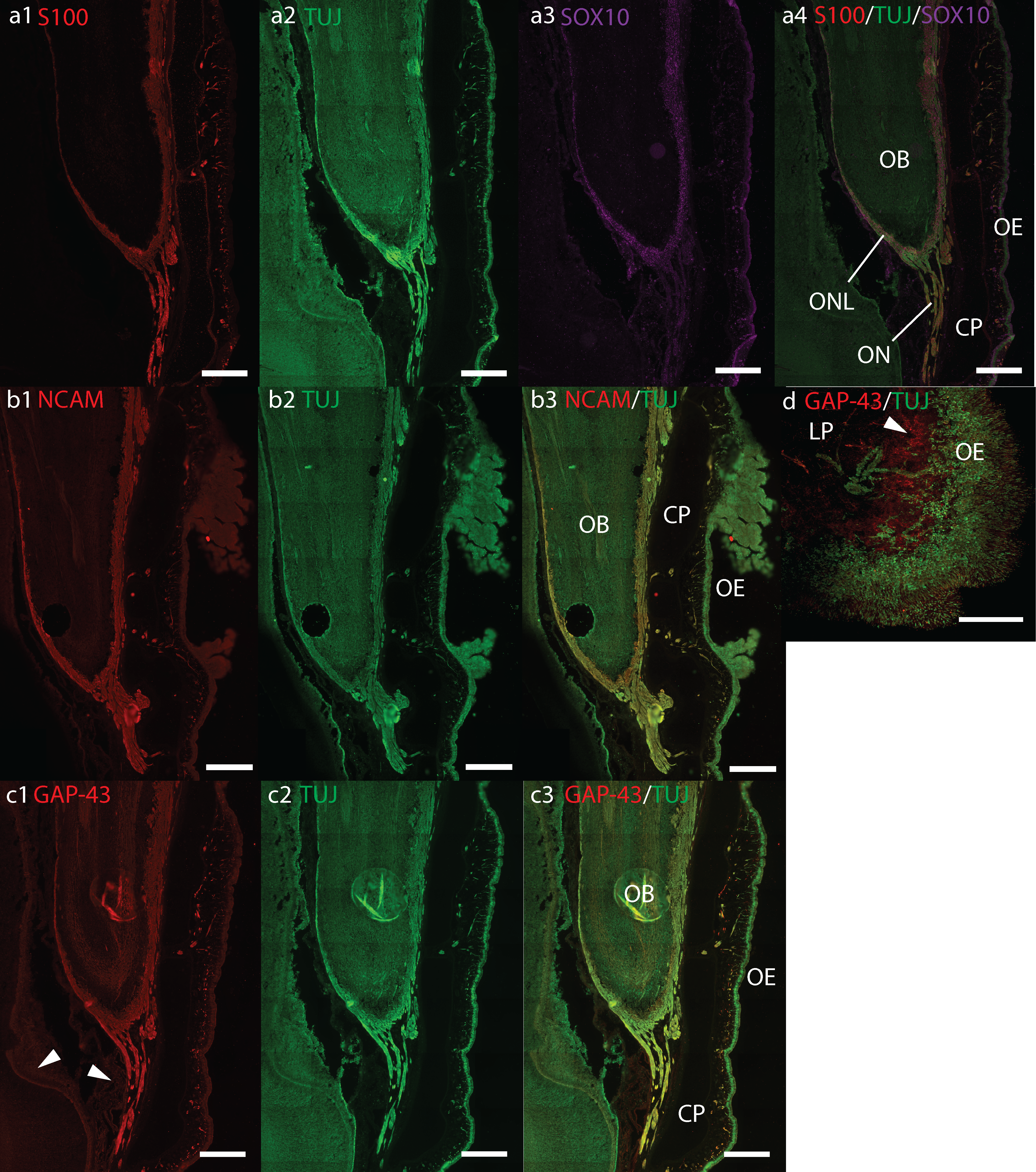

Supplement: Supplementary file 2 — Supplemental Fig. 2 Axioscan fluorescent montages (a-c) and confocal fluorescent micrographs (d) showing sagittal cryosections of 17 pcw human foetal olfactory system immunolabelled with antibodies towards TUJ (green), SOX10 (magenta) and a S100 (red), b NCAM (red), c & d GAP-43 (red). Scale bar a-c 500 μm d 40 μm, CP cribriform plate, LP lamia propria, OE olfactory epithelium, OB olfactory bulb, ONL olfactory nerve layer, ON olfactory nerve. White arrows in c1 and d show weak GAP-43 expression in meninges and under the olfactory epithelium, respectively. (TIFF 29333 kb) [file 429_2016_1313_MOESM2_ESM.tif]

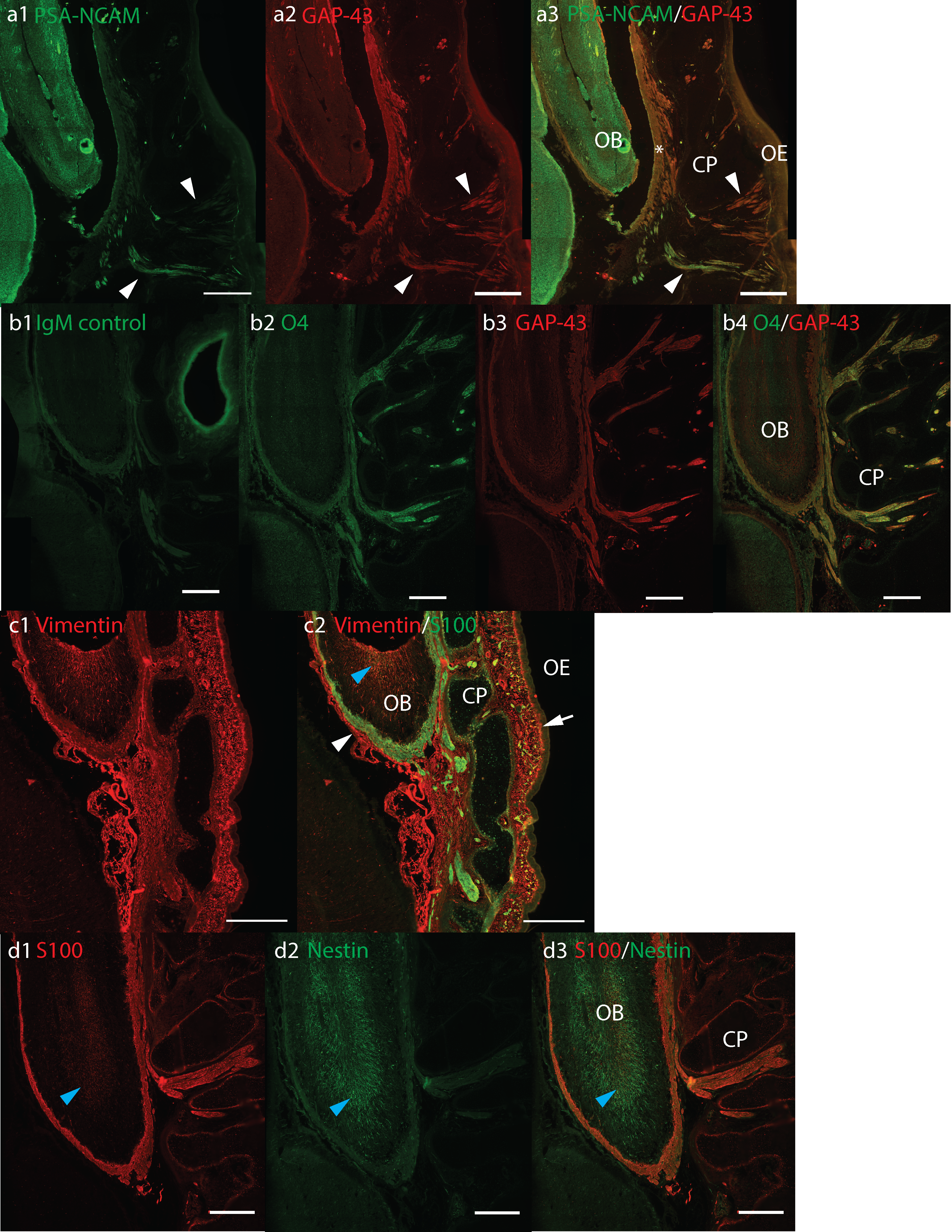

Supplement: Supplementary file 3 — Supplemental Fig. 3 Axioscan fluorescent montages showing sagittal cryosections of a 12 pcw and b-d 17 pcw human foetal olfactory system immunolabelled with antibodies towards a PSA-NCAM (green) & GAP-43 (red), b1 O4 IgM isotype control, b2-b4 O4 (green) & GAP-43 (red), c S100 (green) & vimentin (red), d nestin (green) & S100 (red). Scale bar 500 μm, CP cribriform plate, OE olfactory epithelium, OB olfactory bulb. In a the olfactory nerve layer (*) has peeled away from the OB and adhered to the CP; white arrowheads point to bundles of olfactory nerves showing heterogeneous labelling with PSA-NCAM. c white arrowhead points to vimentin+ meninges, white arrow indicates vimentin in the OE, blue arrowhead shows vimentin/S100+ cells radiating outward from the OB centre. d blue arrowheads point to nestin/S100+ cells radiating outward from the OB centre. (TIFF 30784 kb) [file 429_2016_1313_MOESM3_ESM.tif]

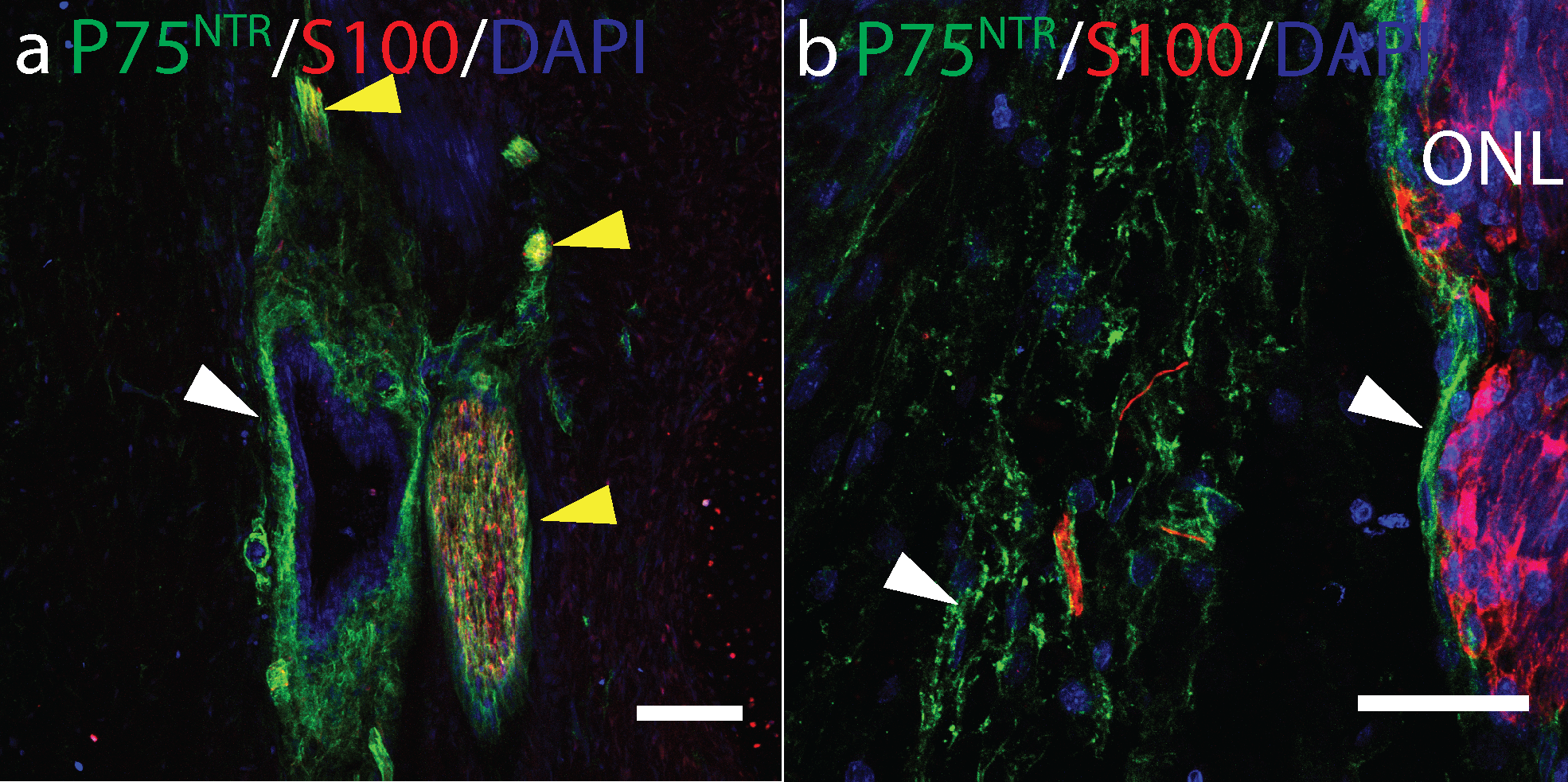

Supplement: Supplementary file 4 — Supplemental Fig. 4 Confocal fluorescent micrographs of sagittal cryosections of 17 pcw human foetal olfactory system immunolabelled with antibodies towards P75NTR (green), S100 (red) and DAPI (blue). a arteriole (white arrow) in the vicinity of olfactory nerves surrounded by large and small peripheral nerve bundles (yellow arrowheads), scale bar 100 μm. b P75NTR+ S100− cells (white arrows) surrounding the outside and covering the surface of the olfactory bulb, scale bar 40 μm. (TIFF 3491 kb) [file 429_2016_1313_MOESM4_ESM.tif]
